# Analysis of Volatile Compounds in *Citri grandis* from Different Regions in South China and the Response of Volatile Compounds to Ecological Factors

**DOI:** 10.3390/molecules30030622

**Published:** 2025-01-31

**Authors:** Shuangfei Hu, Ao Zhang, Hao Wu, Wei Peng, Peibo Li, Weiwei Su

**Affiliations:** Guangdong Engineering and Technology Research Center for Quality and Efficacy Re-Evaluation of Post-Marketed TCM, Guangdong Provincial Key Laboratory of Plant Stress Biology, State Key Laboratory of Biocontrol, School of Life Sciences, Sun Yat-sen University, Guangzhou 510275, China

**Keywords:** authenticity, Huajuhong, immature pomelo, volatile compounds, climatic factors, soil elements, multivariate statistical analysis

## Abstract

*Citri grandis* Exocarpium (Chinese name Huajuhong, HJH) is a traditional Chinese medicinal herb widely used in traditional medicines and foods in China due to its efficacy in treating coughs and excessive phlegm. This study employed HS-SPME-GC-MS to analyze the volatile compounds in HJH samples from different regions, with the aim of distinguishing samples from Huazhou from those of other origins and exploring their potential relationship with ecological factors. A multidimensional strategy was utilized to analyze the relationships between volatile oils, climatic factors, and soil elements, examining how volatile compounds responded to ecological factors. From 47 batches of HJH samples across various regions, eight significantly different volatile compounds were identified, serving as chemical markers for HJH from Huazhou. The findings elucidate the impact of ecological factors on the volatile compounds of HJH, highlighting environmental factors relating to the authenticity of HJH from Huazhou. The results indicate that the authenticity of HJH is shaped by the unique climatic and soil environments of Huazhou.

## 1. Introduction

According to the Chinese Pharmacopoeia (ChP 2020), *Citrus grandis* ‘Tomentosa’ (Huazhouyou) and *Citrus grandis* (L.) Osbeck are legitimate sources of the traditional Chinese medicine material Huajuhong (HJH), which is an important medicinal herb used to treat cough and excessive phlegm [1]. The aroma of HJH is fragrant, and it has a bitter and slightly acrid taste. Given that pomelo is widely distributed in southern China, regions such as Zhejiang, Hunan, Hubei, Jiangxi, Guangxi, and Guangdong are recognized as production regions of HJH. Among these, Huazhou is designated as the authentic production region, while the other locations are classified as non-authentic production regions. HJH cultivated in Huazhou is regarded as the highest-quality authentic herbal medicine [2], commanding a higher market value. This has led to unscrupulous traders using HJH from other regions instead of HJH from Huazhou [3], which adversely affects the safety and efficacy of its clinical application. Therefore, it is imperative to establish reliable methods for distinguishing between authentic and non-authentic HJH to address market discrepancies. Moreover, elucidating the scientific basis of HJH’s authenticity could facilitate the sustainable development of its industrial production.

The principal bioactive compounds of HJH include flavonoids, volatile oils, and other related substances. To date, flavonoid compounds contained in HJH have been analyzed and reported in numerous studies [4,5,6], while volatile compounds have received relatively limited attention. Previous studies have found that volatile substances possess antitussive and expectorant effects [7,8,9]. Notably potent compounds include D-limonene [10], β-pinene [11], γ-terpinene [12], and terpinene-4-ol [13], which exhibit significant antibacterial, anti-inflammatory, antioxidant, and expectorant activities. The volatile compounds of *Citri grandis* primarily consist of terpenes and their derivatives, such as limonene, pinene, and terpinen-4-ol [14]. The variety and abundance of these volatile compounds are crucial in the medicinal efficacy of *Citri grandis* [14]. However, reports on the variation of volatile compounds of HJH from different regions remains limited. Nevertheless, volatile compounds in herbal medicines have demonstrated good performance in distinguishing materials from different regions. For instance, the volatile compounds of mugwort leaves [15] and the volatile constituents of citrus peels can be utilized to differentiate products originating from various species or different geographical regions [16,17]. Volatile compounds possess significant efficacy [18] and as plant secondary metabolites, their composition and abundance are closely correlated with their geographical origin [19]. Therefore, comparative analysis of products from different production regions holds substantial significance.

Environmental factors are one of the key determinants of authenticity, primarily in relation to the geographical origin of herbal medicine, as crucial prerequisites for the accumulation of active components [20]. The growth of medicinal plants is dependent on their environmental conditions, leading to differences in the material composition of medicinal herbs from various production regions [21,22,23,24,25,26]. Differences in climate characteristics and soil physicochemical properties result in variations in the effective component content of medicinal plants [25,26]. For instance, ecological factors in the production region are the key factors affecting variations of the active components in *Salvia miltiorrhiza* [27], with appropriate low temperatures promoting the accumulation of ginsenosides in cells [28,29,30]. The mineral elements in the soil of different production regions also contribute to the accumulation of herbal medicine constituents. For instance, the synthesis and accumulation of monoterpene components in the fruit of *Citrus reticulata* ‘Chachi’ are influenced by soil nutrients and salinity [31]. Soil factors affect the accumulation of active components in herbal medicine [32].

For individual herbal medicines, the genetic mechanisms generally remain consistent in standard agricultural practices. The primary factors that contribute to the authenticity of herbal medicines are predominantly the results of changes in key environmental factors, which subsequently alter the broader ecological context [33]. For example, under drought stress, *Scutellaria baicalensis* can stimulate the expression of key enzyme genes involved in biosynthetic pathways of active components in its roots and leaves [34]. Environmental factors significantly influence the accumulation of effective components in herbal medicines, and the environmental factors influencing HJH vary across different production regions. Ancient Chinese medical books noted that HJH from Huazhou demonstrated enhanced cough-suppressing and expectorant effects, attributed to the rich presence of *Chloriti Lapis* [35] components in the soil where it was cultivated, yet there has been a paucity of research regarding the impact of these factors on its quality. Accordingly, elucidating the authenticity of HJH from an environmental perspective might further enhance the scientific understanding of its authenticity effects.

This study utilized immature pomelo fruits sourced from various regions in China as research materials for sample preparation. It employed headspace solid-phase microextraction gas chromatography–mass spectrometry (HS-SPME-GC-MS) technology for the relative quantitative analysis of volatile substances in samples from different regions. Additionally, ICP-OES was utilized to determine the total concentrations of Al, B, Ca, Cu, Fe, K, Mg, Mn, Na, P, and S in the soil, while climatic factor information was derived according to the geographic coordinates of the sampling points. A chemometric approach was applied to establish a discrimination model for HJH from different production regions, identifying material with variability in quality. By integrating soil and climatic factor data for correlation analysis, this study aimed to elucidate and clarify the influence of these factors on the primary active components in HJH, highlighting the most significant influencing variables. The findings clarify the relationship between ecological factors and the secondary metabolites in HJH, thereby explaining the environmental basis of the authenticity of HJH from Huazhou.

## 2. Results

### 2.1. GC MS Analysis

**Figure 1:**
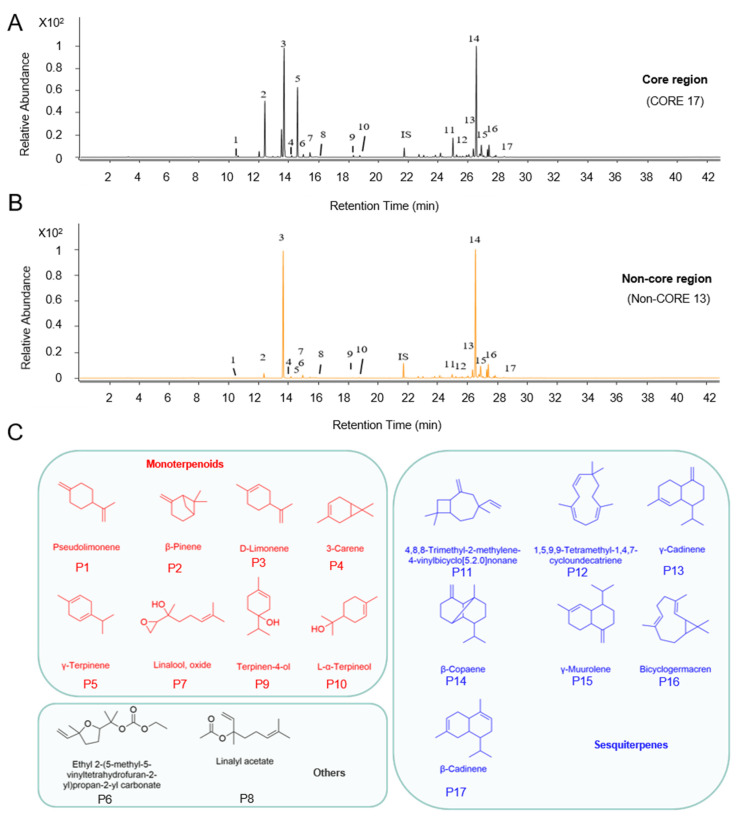
GC-MS typical total ion chromatograms (TICs) of volatile compounds in the pomelo. Comparative analysis of essential oils in the fruit of pumelo in different planting areas: (**A**) TICs of volatile compounds in the HJH from the core region (CORE 17); (**B**) TICs of volatile compounds in the HJH from the non-core region (Non-CORE 13). Numbers represent the common peak order in both COREs and Non-COREs; (**C**) Classification of volatile oil from HJH. Numbers represent the peak order. The peak number represents the common peak in (**A**,**B**).

Volatile compounds were extracted from HJH samples sourced from various origins using solid-phase microextraction (SPME) and subsequently analyzed via gas chromatography–mass spectrometry (GC-MS). The reproducibility of the method was assessed by performing six replicate measurements on a specified sample. The relative standard deviation (RSD) values ranged from 2% to 12%, indicating the method’s good reproducibility (generally close to or lower than 10%) [17]. The acquired results showed that the conditions for extraction and GC-MS analysis were reliable and robust. A total of 33 volatile compounds were identified, primarily consisting of terpene compounds, aligning with findings reported by Fan et al. [14] that identified D-limonene, γ-terpinene, and β-pinene as the predominant constituents of HJH essential oil. Notably, m-cymene was identified as a unique component in COREs; detailed compound information is available in Table 1. Figure 1A,B illustrate the total ion chromatograms (TICs) of essential oils from COREs compared with those from Non-COREs, revealing that COREs contained a greater diversity of compounds. Further characterization of these components revealed 12 monoterpenes, 19 sesquiterpenes, as well as two ester compounds (results depicted in Table 1). Monoterpenes, such as D-limonene, are classified as compounds composed of two isoprene units, typically containing ten carbon atoms, with the fundamental carbon skeleton formed by head-to-tail sequence connection of isoprene units. Oxygenated derivatives of monoterpenes, such as compounds 11, 13, and 14, were also present. In contrast, sesquiterpenes consist of three isoprene units and contain fifteen carbon atoms, with γ-cadinene illustrated as an example of this class, exhibiting a similar head-to-tail connection of isoprene units.

From the mass spectrometric data of 47 samples from different origins, 17 common peaks were selected (Figure 1C), encompassing the principal compounds in HJH essential oil. The relative peak areas of these compounds were compiled into a dataset, including the specific data detailed in Table 2; the average sum of the relative peak areas accounted for 88.37%. Studies have shown [36,37] that solid-phase microextraction (SPME) can also be used for quantitative analysis in a non-equilibrium state. Therefore, as long as the samples are extracted under the same conditions, the differences in volatile compounds of HJH samples from different regions can be compared. Research indicates that the compounds represented by common peaks, as well as the peaks with higher relative abundances, demonstrate faster identification rates, enhanced stability, and improved quantitative repeatability [38]. Accordingly, the analysis excluded certain minor or trace volatile compounds.

**Table 1:** Detailed compound information.

No.	RT/min	Compound	Formula	CAS No.	*RI*	Reference	Sim Score
1	10.56	α-Pinene	C_10_H_16_	80-56-8	930	[39]	94.73
2	11.97	Pseudolimonene	C_10_H_16_	499-97-8	975	[40]	95.35
3	12.36	β-Pinene	C_10_H_16_	127-91-3	988	[41]	96.21
4	12.89	α-Phellandrene	C_10_H_16_	99-83-2	1004	[42]	91.13
5	13.48	m-Cymene	C_10_H_14_	535-77-3	1023	[43]	97.13
6	13.65	D-Limonene	C_10_H_16_	5989-27-5	1029	[44]	98.35
7	14.15	3-Carene	C_10_H_16_	13466-78-9	1045	[40]	95.09
8	14.55	β-Terpinene	C_10_H_16_	99-85-4	1058	[45]	97.75
9	14.93	Ethyl 2-(5-methyl-5-vinyltetrahydrofuran-2-yl) propan-2-yl carbonate	C_13_H_22_O_4_	-	1048	[46]	95.8
10	15.38	α-Terpinene	C_10_H_16_	99-86-5	1084	[45]	95.51
11	15.43	Linalool oxide	C_10_H_18_O_2_	1365-19-1	1086	[47]	92.93
12	15.82	Linalyl acetate	C_12_H_20_O_2_	115-95-7	1093	[48]	80.05
13	18.29	Terpinen-4-ol	C_10_H_18_O	562-74-3	1178	[42]	93.14
14	18.72	α-Terpineol	C_10_H_18_O	10482-56-1	1191	[49]	94.51
15	22.68	δ-EIemene	C_15_H_24_	20307-84-0	1410	[39]	95.33
16	23	Cadina-3,5-diene	C_15_H_24_	267665-20-3	1416	[46]	94.22
17	23.23	Humulene	C_15_H_24_	6753-98-6	1421	[50]	85.33
18	23.78	Copaene	C_15_H_24_	3856-25-5	1433	[41]	96.18
19	24.12	Guaia-10(14),11-diene	C_15_H_24_	-	1440	-	95.09
20	24.95	2-methylene-4,8,8-trimethyl-4-vinyl-bicyclo[5.2.0]nonane	C_15_H_24_	-	1458	-	97.36
21	25.43	Valencene	C_15_H_24_	4630-07-3	1469	[51]	94.78
22	25.86	1,5,9,9-Tetramethyl-1,4,7-cycloundecatriene	C_15_H_24_	-	1478	[52]	94.77
23	26.02	Bicyclosesquiphellandrene	C_15_H_24_	54324-03-7	1481	[53]	94.8
24	26.32	γ-Cadinene	C_15_H_24_	483-74-9	1488	[50]	96.69
25	26.52	β-Copaene	C_15_H_24_	18252-44-3	1492	[42]	97.35
26	26.76	γ-Muurolene	C_15_H_24_	30021-74-0	1497	[48]	95.3
27	26.86	Bicyclogermacren	C_15_H_24_	67650-90-2	1499	[54]	95.28
28	27.01	δ-Cadinene	C_15_H_24_	483-76-1	1502	[50]	92.8
29	27.37	β-Cadinene	C_15_H_24_	523-47-7	1510	[55]	94.82
30	27.46	Calamenene	C_15_H_24_	72937-55-4	1512	[46]	93.19
31	27.73	4-Isopropyl-1,6-dimethyl-1,2,3,4,4a,7-hexahydronaphthalene	C_15_H_24_	16728-99-7	1518	[56]	86.44
32	27.83	α-Amorphene	C_15_H_24_	483-75-0	1520	[57]	94.92
33	28.4	Germacrene B	C_15_H_24_	15423-57-1	1532	[39]	91.84
IS	21.69	n-Tridecane	C_13_H_28_	629-50-5	1300	-	96.59

‘’RT’’, retention time (min). “-” indicates a compound without a CAS number. “*RI*”, retention index.

### 2.2. Discrimination Between COREs and Non-COREs Based on Multivariate Analysis

**Figure 2:**
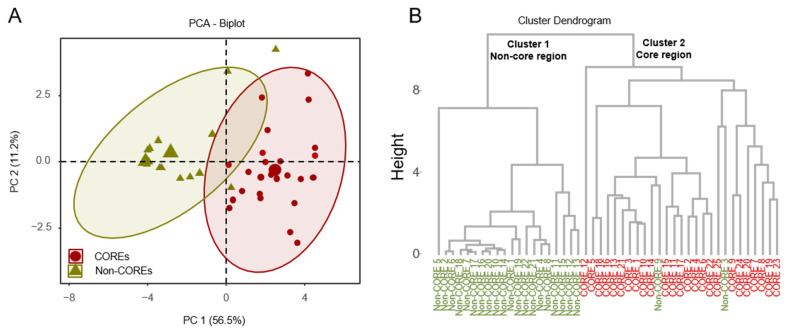
Comparative analysis of essential oils in pomelo fruit collected from different regions: (**A**) Principal component analysis (PCA) of Euclidean distance for the essential oil contents in core and the non-core region samples; (**B**) Dendrograms of the hierarchical cluster analysis (HCA) result. Red points mean COREs. Olive green points mean Non-COREs. Red fond means COREs, and olive green fond means Non-COREs.

This study employed principal component analysis (PCA) to investigate potential differences between COREs and Non-COREs. Upon determining the projections for each principal component, each sample was assigned a score based on these components. A PCA score plot was generated using the scores PC1 and PC2 from the two principal components, as illustrated in Figure 2A. In the presented study, four principal components accounting for 82.4% (R^2^X) of the total variance were considered significant (PC1 described 56.5% of the sample variability, and PC2 described 11.2%). The results indicated that PC1 had the highest cumulative contribution rate to the total variance, and the predictive ability of the model (Q^2^) was 0.508, suggesting that the model required further optimization (Q^2^ ≥ 0.50). The score plot enables observation of the degree of clustering and dispersion among samples; closer distribution points indicate greater similarity, while greater separation suggests significant differences. In the plot, the COREs and Non-COREs are located within distinct regions, with a noticeable separation between them, indicating significant differences in volatile compounds between the samples from the two groups. Comparing the score plot and the loading plot, differential intra-group or inter-group compounds could be rapidly identified. The significance of the differentiation was validated through statistical analyses, including *t*-tests and ANOVA.

Additionally, hierarchical clustering was performed using the Ward method, generating a dendrogram as illustrated in Figure 2B. The results indicate that the 47 samples could be broadly categorized into two groups: COREs and Non-COREs, highlighting a clear distinction in volatile compounds between HJH from Huazhou and samples from other regions.

**Figure 3:**
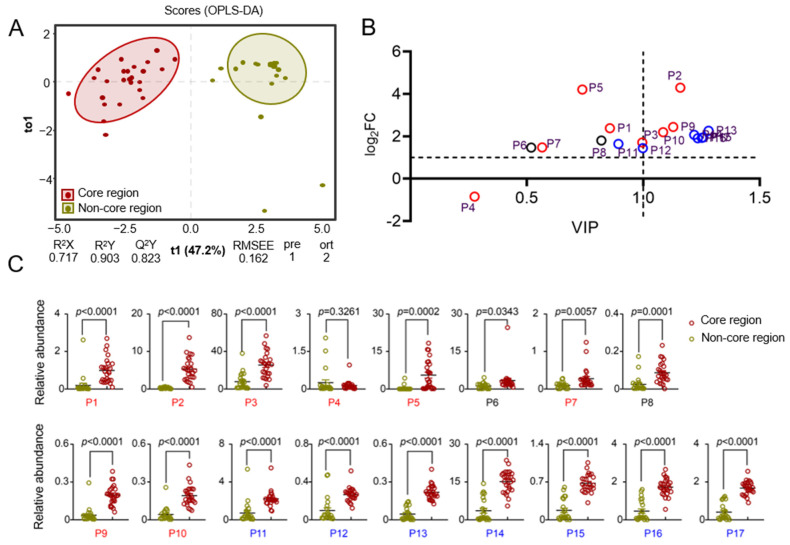
Screening of volatile compound quality markers across samples from different regions: (**A**) Score plot of orthogonal partial least squares discrimination analysis (OPLS-DA) models for the classification of pomelo; (**B**) Quadrant plot constructed using the Variable Importance in Projection (VIP) values obtained from OPLS-DA as the *x*-axis, while the *y*-axis represents the Log_2_FC values of the relative abundance of volatile compounds. Compounds with VIP values greater than 1 and Log_2_FC values greater than 1 were selected as potential quality markers; (**C**) Results of *t*-tests on the relative abundance of compounds across different regions. P1, pseudolimonene; P2, β-pinene; P3, D-limonene; P4, 3-carene; P5, γ-terpinene; P6, ethyl 2-(5-methyl-5-vinyltetrahydrofuran-2-yl)propan-2-yl carbonate; P7, linalool oxide; P8, linalyl acetate; P9, terpinen-4-ol; P10, α-terpineol; P11, 2-methylene-4,8,8-trimethyl-4-vinyl-bicyclo[5.2.0] nonane; P12, 1,5,9,9-tetramethyl-1,4,7-cycloundecatriene; P13, γ-cadinene; P14, β-copaene; P15, γ-muurolene; P16, bicyclogermacren; P17, β-cadinene. Red represents monoterpenes, blue represents sesquiterpenes, and black represents other types of compounds in Figure 3B.

To achieve more precise results, the mass spectrometry data of COREs and Non-COREs were evaluated based on supervised OPLS-DA. The OPLS-DA results for the volatile oil components of HJH indicated a clear separation between the authentic and non-authentic regional samples (see Figure 3). The scores for the first principal component (horizontal axis) positioned the COREs predominantly on the negative half of the axis, while the Non-COREs were situated on the positive half of the axis, suggesting significant differences in volatile oil composition based on geographical origin. The model identified one predictive component and two orthogonal components. The fitting index for the independent variables (R^2^X) was 0.717, indicating that the three principal components accounted for 71.7% of the variance in the X variable, with the predictive component contributing 47.2% and the orthogonal components contributing 24.5%. The fitting index for the dependent variable (R^2^Y) was 0.903, signifying that the predictive component accounted for 90.3% of the variance in the Y variable. In addition, the model’s predictive index (Q²) was 0.823, demonstrating 82.3% prediction accuracy for different origins of HJH. Both R² and Q^2^ exceeded 0.5, indicating a good model fit. In the permutation test results, the R^2^Y and Q^2^Y values of the actual and simulated models, after random permutation, yielded scatter plots where the model R^2^Y and Q^2^Y (scatter) values were both lower than the true values (indicated by the horizontal line). Additionally, the p-values for the R²Y fitting index and the Q^2^ predictive index were both equal to 0.05, suggesting that the model fitted well without overfitting. The variable contribution within the OPLS-DA model was assessed based on the Variable Importance for the Projection (VIP) score, defined as the weighted sum of the PLS weights. Variables with a VIP score greater than 1 were considered significant in the model. Moreover, the Log_2_FC for each compound was calculated based on its relative abundance. Components with both VIP and Log_2_FC values greater than 1 were selected as potential quality markers, resulting in a total of eight components (α-terpineol (P10), γ-cadinene (P13), β-copaene (P14), γ-muurolene (P15), bicyclogermacren (P16), β-cadinene (17), β-pinene (2), and terpinen-4-ol (P9)). These eight compounds exhibited significant differences in content between COREs and Non-COREs (*p* < 0.001), indicating their potential as distinguishing biomarkers.

### 2.3. Environmental Factors Analysis

**Figure 4:**
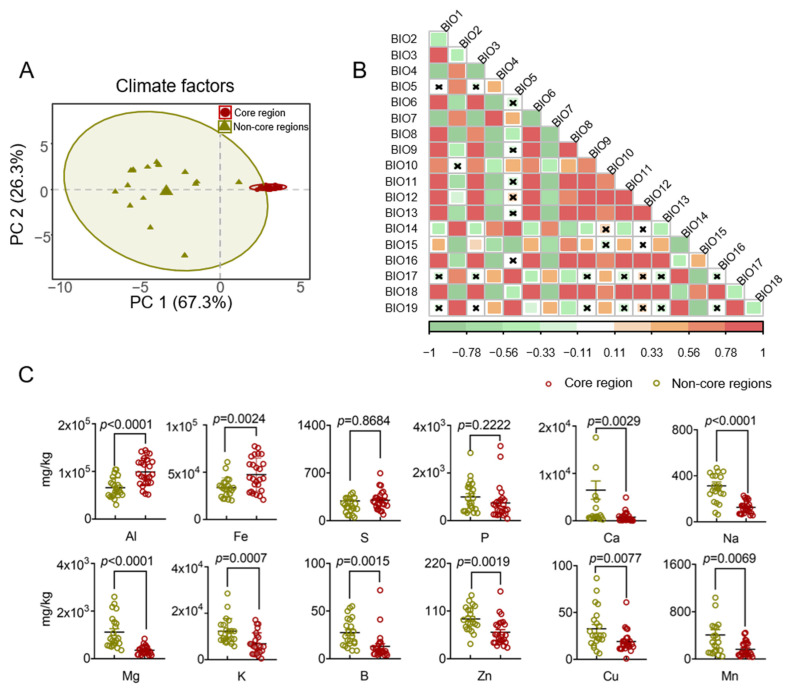
Comparative analysis of environmental factors in different pomelo-collecting regions: (**A**) Principal component analysis of Euclidean distance for climatic factors in core and non-core region samples; (**B**) Auto-correlation of climatic factors by Pearson analysis; (**C**) Soil physical and chemical properties in rhizosphere soil samples from the core region differed from those in samples from the non-core region. Significance between core and non-core regions indicated by *t*-test. Red means core regions, and olive green means non-core regions.

The synthesis and accumulation of plant secondary metabolites are influenced not just by species, organ, and growth developmental stages but also by ecological factors [58]. The concentration of secondary metabolites in plants is correlated with climatic and soil conditions in their respective regions. To investigate the impact of different cultivation areas on the accumulation of volatile compounds in HJH, it is essential to ascertain whether ecological factors differ across various production regions. The results indicate that the climatic conditions in the core and non-core regions are significantly distinct (Figure 4A). For the first principal component (PC1), the climatic factors of the core region predominantly clustered on the positive side, while those of the non-core region were mainly positioned on the negative side. This suggests that geographic factors were the primary contributors to the observed climatic differences, with an explanatory power of 67.3% for PC1. Moreover, PCA dimensionality reduction analysis revealed that soil factors from different regions could generally be grouped into two categories (Figure 4C). For the first principal component (PC1), the soil factors of the core region were primarily located on the negative side of the horizontal axis, whereas those from the non-core region clustered on the positive side. Similarly to climatic factors, regional differences emerged as a significant influence on soil element variation, accounting for 36.8% of the explanatory power. These results underscore the evident differences in climatic and soil factors between the core and non-core regions, with the variability in climatic factors being more pronounced than that in soil factors, suggesting that they may be crucial determinants of volatile oil content in HJH.

To address the issue of multicollinearity among climate factor variables, the selection of variables (temperature, temperature change, precipitation, precipitation change, and soil) was carried out based on distinct climate factor categories. Environmental factors with similar characteristics and low contribution rates were excluded. In this study, four principal components accounting for 98.3% (R^2^X) of the total variance were considered significant (PC1 described 67.3% of the sample variability, and PC2 described 26.3%). The predictive ability of the model (Q^2^) was 0.956, which indicated that it was a good model (Q^2^ ≥ 0.50). Both domains (core region and non-core region) were clearly separated from each other. Through the integration of PCA and Pearson correlation analysis, environmental factors with absolute loadings exceeding 0.80 in the PCA were identified. In cases where two or more correlation coefficients ∣r∣ ≥ 0.8 were observed among the same environmental factors, the factor with the highest contribution rate was chosen to differentiate between the two regions [59]. The soil factors were selected to include differences between the two groups and contain statistically significant elements. The screening results are presented in Table 3.

### 2.4. Correlation Analysis of Climatic Factors and Soil Factors with Volatile Compounds

**Figure 5:**
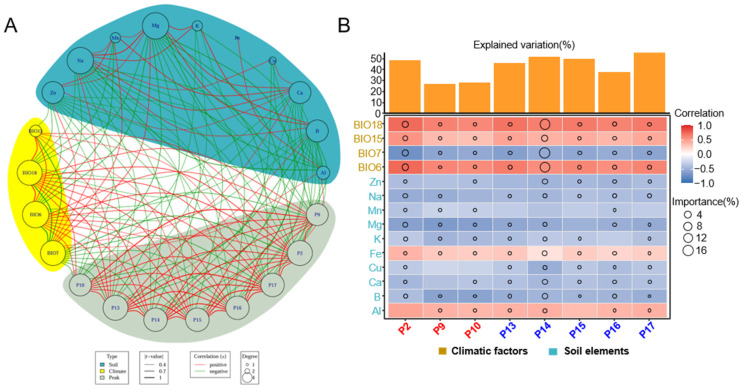
Correlation analysis of soil and climate elements with volatile compounds in pomelos sourced from core and non-core regions: (**A**) A network was constructed by correlation of compounds (grey), climatic factors (yellow), and soil elements (green). Node size corresponds to the degree of each node. The thickness and color of the edges denote strength and significance, respectively. Red and green lines indicate positive and negative correlations; (**B**) Contributions of soil physical–chemical properties and climatic factors to the components with varying levels among different regions in plants. P 10, α-terpineol; P13, γ-cadinene; P14, β-copaene; P15, γ-muurolene; P16, bicyclogermacren; P17 β-cadinene; P2, β-pinene; P9, terpinen-4-ol.

Plants produce a variety of secondary metabolites to adapt to diverse environmental stresses during their growth. These secondary metabolites often possess multiple biological activities, providing humans with healthy food and medicinal products [60]. In this study, a correlation analysis was conducted to examine the relationship between the volatile compounds content from HJH of different origins characterized by various climatic factors and soil mineral elements (Figure 5A). Specifically, BIO15, BIO18, and BIO6 exhibited a positive correlation with the volatile oil content of HJH, while BIO7 showed a negative correlation. Furthermore, BIO18 was negatively correlated with sodium (Na), manganese (Mn), and magnesium (Mg) in the soil, whereas BIO15 had a negative correlation with zinc (Zn) content. Additionally, BIO6 demonstrated negative correlations with Zn, Na, Mn, and Mg. Conversely, BIO7 was positively correlated with the concentrations of Zn, Na, Mn, Mg, and aluminum (Al) in the soil. The soil boron (B) content was negatively correlated with BIO6 and BIO18 but positively correlated with BIO7. B was positively correlated with Na, Mg, and potassium (K), while calcium (Ca) was positively associated with Zn, Na, Mn, Mg, and copper (Cu). Copper (Cu) showed a positive correlation with Na, and Al was positively correlated with iron (Fe). Notably, Al was also positively correlated with the volatile oil component P9. The volatile oil components α-terpineol (P10), γ-cadinene (P13), β-copaene (P14), γ-muurolene (P15), bicyclogermacren (P16), β-cadinene (P17), β-pinene (P2), and terpinen-4-ol (P9) exhibited positive correlations with the climatic factors BIO15, BIO18, and BIO6, while demonstrating negative correlations with BIO7 and the soil elements Zn, Na, Mg, and Ca.

The analysis was conducted through multiple linear regression and variance decomposition (Figure 5B). The results revealed that the selected climatic and soil factors collectively explained 48.62% of the variance in β-pinene (P2), 26.58% in terpinen-4-ol (P9), 28.13% in α-terpineol (P10), 45.59% in γ-cadinene (P13), 51.55% in β-copaene (P14), 49.63% in γ-muurolene (P15), 37.55% in bicyclogermacren (P16), and 55.64% in β-cadinene (P17) (Figure 5B). Notably, the climatic factors—Precipitation in the Warmest Quarter (BIO18), Precipitation Seasonality (BIO15), Precipitation in the Driest Quarter (BIO7), and Precipitation in the Wettest Quarter (BIO6), as well as the concentrations of B, Mg, Ca, Zn, and Fe in the soil, were identified as key determinants for distinguishing material with potential variability in quality across different regions. The influence of climatic factors, particularly concerning the variability of β-copaene (P14), was notably pronounced, showing a positive correlation with BIO18, BIO15, BIO6, and soil Fe, while displaying a negative correlation with soil concentrations of Ca, B, and Zn. In comparison to climatic factors, the mineral element in the soil had less influence on the differences in the content of potential quality differentiators in HJH, and the concentrations of Fe and Al in the soil exhibited a positive correlation with the levels of material with potential variability in quality, whereas other elements generally correlated negatively.

**Figure 6:**
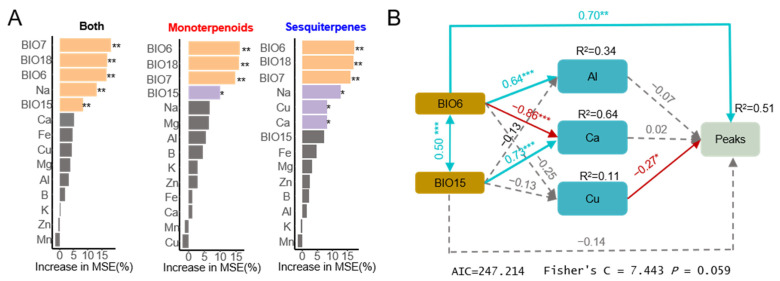
The major factors influencing volatile compounds evaluated by Random Forest and SEM analysis: (**A**) The main factors influencing the relative abundance of compounds were analyzed and evaluated based on a random forest model. Both means the sum of the relative abundances of monoterpene compounds and sesquiterpene compounds. The monoterpenes part represents the average relative abundance of monoterpene compounds included in the model. The sesquiterpenes part represents the average relative abundance of sesquiterpene compounds included in the model; (**B**) The direct and indirect relationships between climatic factors, soil elements, and the total relative abundance of compounds, assessed based on structural equation modeling (SEM). The arrows represent the direction of hypothesized causation. The red line represents adverse effects, the blue line represents positive effects, and the gray line represents no significant effect. * *p* < 0.05, ** *p* < 0.01 and *** *p* < 0.001. In this part, “peaks” means the sum result of the relative peak areas of the eight selected compounds.

A random forest analysis was employed to further identify the factors associated with the volatile components in traditional medicines, with the results presented in Figure 6A. BIO7, BIO15, and BIO18 were identified as the primary factors influencing monoterpenes and sesquiterpenes. The main influencers of monoterpene content included BIO6, BIO7, BIO15, and BIO18, while the key factors impacting sesquiterpene content included BIO6, BIO7, BIO18, Na, Cu, and Ca. Additionally, considering the geological characteristics of the core region, factors such as Al and Fe (elements that are primarily found in *Chloriti Lapis*, which is rich in the soil of Huazhou ) were included in the subsequent SEM validation model. In the SEM model constructed in this study, a *p*-value less than 0.05 indicated that the model exhibited a good fit to the data and was suitable for data validation. The outcome showed that BIO6 had a positive correlation with the abundance of volatile compounds, whereas Cu content in soil was negatively correlated with the abundance of volatile compounds. The results indicate that the unique climate in the core region and soil elements together affect the volatile compounds in medicinal materials.

## 3. Discussion

This study employed HS-SPME-GC-MS technology for the relative quantification of volatile compounds in the COREs and Non-COREs. Subsequently, OPLS-DA was conducted to identify variable-quality material in the essential oils from different regions. Permutation tests indicated a good fit for the model, with no evidence of overfitting, suggesting that the identification of materials with potential variability in quality was statistically significant. The volatile compounds in HJH are known to have expectorant and antitussive effects [7,8,9]. Based on their chemical structures, these compounds can be categorized into three main types: monoterpenoids, sesquiterpenoids, and a small amount of other compounds. The monoterpenoid compounds include pseudolimonene, β-pinene, D-limonene, 3-carene, γ-terpinene, linalool oxide, terpinen-4-ol, and α-terpineol. The sesquiterpenoid compounds mainly comprise γ-cadinene, β-copaene, γ-muurolene, bicyclogermacrane, β-cadinene, 2-methylene-4,8,8-trimethyl-4-vinyl-bicyclo[5.2.0]nonane, and 1,5,9,9-tetramethyl-1,4,7-cycloundecatriene. Other compounds include ethyl 2-(5-methyl-5-vinyltetrahydrofuran-2-yl) propan-2-yl carbonate and bicyclo[5.2.0]nonane, as well as linalyl acetate. Currently, there are few studies on the biological activities of Pseudolimonene; however, as a monoterpenoid, it may exhibit potential antimicrobial and antioxidant properties [61]. β-Pinene has demonstrated antimicrobial and antifungal activities, as well as anti-inflammatory and analgesic effects [11]. D-Limonene, the most abundant component in Citrus reticulata volatile oils, exhibits significant antioxidant, antimicrobial, anti-inflammatory properties, along with notable expectorant and antitussive effects [10,61]. 3-Carene shows both antimicrobial and antioxidant activities [62], while γ-terpinene is effective in scavenging free radicals and offers analgesic and expectorant effects [12]. Both terpinen-4-ol and α-terpineol exhibit antimicrobial, anti-inflammatory, and antioxidant activities [13]. Among the sesquiterpenoids, γ-cadinene demonstrates antimicrobial and antioxidant effects. Studies have shown its ability to inhibit the growth of various bacteria and fungi [61]. β-Copaene, γ-muurolene, bicyclogermacrane, and β-cadinene all exhibit antimicrobial and antioxidant activities as well. Additionally, linalool oxide and linalyl acetate not only have anti-inflammatory and antioxidant effects but also inhibit the growth of various bacteria and fungi [63]. While there has been limited research on the biological activities of ethyl 2-(5-methyl-5-vinyltetrahydrofuran-2-yl) propan-2-yl carbonate, 2-methylene-4,8,8-trimethyl-4-vinyl-bicyclo[5.2.0]nonane, and 1,5,9,9-tetramethyl-1,4,7-cycloundecatriene, their chemical structures suggest that they may possess potential antimicrobial and antioxidant properties [61]. These volatile compounds play an important role in the pharmacological activity of traditional Chinese medicine, with D-limonene [10], β-pinene [11], γ-terpinene [12], and terpinen-4-ol [13] being among the most potent and abundant compounds in HJH volatile oils. In conclusion, volatile compounds play a significant role in the pharmacology of traditional Chinese medicine, and drying treatments do not affect their biological activities. This provides a scientific foundation for the development and application of traditional Chinese medicine.

GC-MS data analysis revealed differences in the relative abundance of volatile compounds between the COREs and Non-COREs. Eight potential chemical markers were identified, including α-terpineol (P10), γ-cadinene (P13), β-copaene (P14), γ-muurolene (P15), bicyclogermacrane (P16), β-cadinene (P17), β-pinene (P2), and terpinen-4-ol (P9). Among these, β-pinene, γ-cadinene, terpinen-4-ol, and α-terpineol are known to have significant antitussive and expectorant effects [11,12,13,64,65]. The other four compounds, which have structures similar to those of the aforementioned compounds, may also promote similar effects. Notably, all eight compounds were found to be present in higher concentrations in the COREs, which could be one of the reasons for the superior therapeutic efficacy of authentic medicinal materials.

Furthermore, ICP-OES was utilized to determine the concentrations of Al, B, Ca, Cu, Fe, K, Mg, Mn, Na, P, and S in the soil. Climate factor information was obtained based on the latitude and longitude of sampling points. Significant climate and soil factors distinguishing authentic from non-authentic regions were identified through PCA dimensionality reduction and Pearson correlation tests. Ultimately, correlation analyses between the abundance of volatile compounds in HJH and the soil elements and climatic factors revealed that climatic factors exerted the greatest influence on the volatile compounds, followed by soil elements.

Currently, the recognized functions of volatile compounds in plants generally include disease resistance, pest resistance, and tolerance to environmental stressors [66]. The volatile compounds in HJH are predominantly terpenoids, which play significant defensive roles in plant growth [67,68]. This research indicates that the volatile compounds from authentic HJH-producing regions exhibit greater diversity compared with those from non-authentic regions, with some compounds present in higher abundance. Furthermore, the formation of authentic HJH is closely linked to climatic factors, characterized by relatively high average temperatures, minimal annual temperature fluctuations, and significant yet variable precipitation, indicating instability and frequent abnormal weather patterns. These climatic features relate to Huazhou’s geographical location; situated at latitudes of 21°29′–22°13′ N and longitudes of 110°20′–110°45′ E, it falls within a subtropical climate zone with mild temperatures and abundant rainfall influenced by maritime conditions, leading to frequent summer and autumn typhoons and heavy rainfall. Meanwhile, studies suggest that the biosynthesis of secondary metabolites in plants is related to environmental temperature [23,69,70,71]. Investigations into the volatile compound content of HJH have demonstrated that increasing temperatures correlate with higher abundance of volatile substances, potentially due to the higher temperatures promoting the biosynthesis of volatile compounds [72]. However, reports also indicate that secondary metabolites may decrease under extreme temperature [22]. Temperature is a critical ecological factor influencing community distribution, not only providing the necessary heat for the growth of medicinal plants but also altering the climatic environment of these plants, thereby affecting the production and allocation of secondary metabolites through various mechanisms [73]. Research has found that temperature is a primary factor influencing the release rates of monoterpenes and other volatile compounds, with release rates increasing with temperature within a certain range, probably due to changes in vapor pressure [74,75,76,77]. Additionally, variations in precipitation patterns affect biomass accumulation and allocation to reproductive organs, leading to changes in reproductive strategies that enhance plants’ adaptability to their environments [78]. The above indicates that climatic factors are one of the key factors influencing the authenticity of HJH.

**Figure 7:**
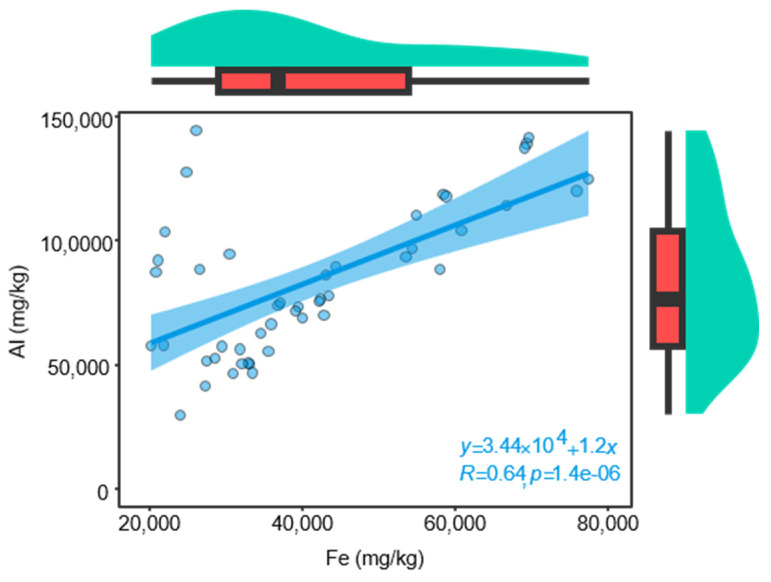
Pearson Correlation analysis of Fe and Al elements in soil. Green shows the data’s kernel density, red represents the data distribution in the box plot, and the blue area indicates the 95% confidence interval.

The soil environment is another one of the key factors influencing plant growth [79], particularly for medicinal herbs. The quality of authentic herbal medicines is strongly dependent on environmental conditions, and the environmental conditions are largely determined by the soil. For instance, soil nutrients have been shown to significantly enhance quality accumulation in herbal medicines such as *Citrus reticulata* ‘Chachi’ [31], *Chrysanthemum morifolium* Ramat. [80], and *Cinnamomum migao* endemic [81]. This enhancement may stem from the influence of various factors, including climate and soil nutrients, on the absorption and accumulation of elements by plants [81]. Soil serves as the fundamental substrate for medicinal plants, and soil nutrients are critical determinants of growth rate and the abundance of active ingredients [82,83]. It is recorded in ancient Chinese medical books that the soil in Huazhou is rich in the mineral component *Chloriti Lapis* (Chinese name Mengshi), a traditional medicinal mineral primarily utilized for its cough-suppressing and phlegm-relieving properties [84], which aligns with the medicinal efficacy of HJH. The primary elements present in *Chloriti Lapis* include Al, Fe, Si, Mg, Ca, K, and Na, which together account for approximately 90.70% [35]. According to Zhonghua Bencao, *Chloriti Lapis* is notably abundant in Fe and Al, consistent with the high concentrations of these elements observed in the soil of Huazhou. In this study, the concentrations of Fe and Al in soil samples from authentic regions were significantly higher than those from non-authentic regions, and a strong positive correlation (r = 0.64) was found between the concentrations of Fe and Al (As shown in Figure 7). Experimental results indicated that the levels of Fe and Al in the soil were positively correlated with the volatile compound content in the HJH samples. This correlation suggests that the unique soil environment of Huazhou contributes to the distinctive quality of the essential oils derived from HJH in this region. Furthermore, among climatic factors, temperature and precipitation have substantial effects on the weathering, leaching, and deposition of substances in the soil [85]. The results of this study indicate that the concentrations of Al and Fe in the soil from authentic regions are higher than those in non-authentic regions. Conversely, levels of other elements such as B, Ca, Cu, K, Mg, Mn, Na, and P are significantly lower in the authentic regions. A positive correlation was found between the volatile compound content in HJH samples and the soil concentrations of Al and Fe, while a negative correlation was observed with the concentrations of B, Ca, Cu, K, Mg, Mn, Na, and P. This phenomenon may be attributed to the subtropical climate of Huazhou, characterized by intense weathering and leaching processes that lead to substantial loss of easily soluble mineral elements, while sparingly soluble elements like Al and Fe remain in the soil, contributing to the reddish color of the soil. This characteristic is consistent with the predominantly reddish hue observed during sampling. The climatic conditions in Huazhou result in low levels of soluble inorganic nutrients, significantly reducing the availability of elements that can be effectively utilized by plants. This situation may induce stress in plants, consequently leading to a measurable increase in the abundance of volatile compounds in HJH. The relative increase in the concentrations of Fe and Al in the soil may represent an external manifestation of soil element loss. In summary, the relatively high concentrations of Fe and Al elements in the soils of the Huazhou region are likely to have been influenced by both geological and climatic factors. The unique soil characteristic may be one of the key determinants shaping the quality of the volatile oils in HJH.

The climatic factors and soil elements of medicinal plant habitats exert varying degrees of influence on the quality of herbal medicines. A deficiency in mineral elements within the soil can affect plant growth and the formation of secondary metabolites. According to the resource availability hypothesis, when a plant’s potential growth rate decreases, the production of secondary metabolites for defense increases [86]. The growth-differentiation balance hypothesis assumes that under conditions of abundant resources, plants prioritize growth; however, under poor resource conditions, growth and differentiation are both reduced. At moderate resource levels—such as mild drought, or habitats with warm or cold temperatures—differentiation becomes the primary focus, accompanied by greater accumulation of secondary metabolites [86]. It has been reported that the soil environment shapes unique rhizosphere microbial communities, which in turn contribute to the authenticity of herbal medicines [31]. In this study, the soil element content was found to influence the levels of volatile compounds in HJH. The total concentrations of elements such as Al, B, Ca, Cu, Fe, K, Mg, Mn, Na, P, and S were measured, but without separately assessing the plant-available valence states of these elements. Research indicates that plants can utilize soil elements not only directly but also indirectly through microorganisms [31,87]. This might be one of the complex factors relating to the authenticity of herbal medicines, warranting further investigation into how plants absorb and utilize soil elements. It should be noted that this study focused solely on the total concentration of elements in the soil without addressing their bioavailability, which has a significant effect on absorption in plants. However, the bioavailability of elements in soil involves multiple influencing factors, including soil physicochemical properties, soil fertility, and soil microecology [22,31]. Our group will continue to work on this topic.

## 4. Materials and Methods

### 4.1. Subsection

Field sampling was carried out between May and July 2023. In this study, to ensure consistency and comparability of the experiments, all immature pomelo fruit samples were harvested at a standardized time exactly two months after flowering. This standardized harvesting schedule aimed to eliminate potential variability caused by different harvesting times, thereby ensuring the accuracy and reliability of the experimental results. A total of 47 batches of fruit samples and soil samples (10–20cm) from around fruit trees were selected from sites in seven provinces of China (Guangdong, Guangxi, Hunan, Sichuan, Guizhou, Jiangxi, and Zhejiang). The latitude and longitude information at the sampling points was recorded using an Element Latitude Camera. Sampling sites were categorized based on their geographical region, into authentic producing areas (specifically Huazhou in Guangdong Province) and non-authentic producing areas (other regions), designated as core regions and non-core regions, respectively. Corresponding samples were recorded as COREs and Non-COREs.

In total, 47 batch samples of immature pomelo fruits from various regions were collected (22 Non-COREs and 25 COREs). These samples were authenticated by Professor Wen-bo Liao at Sun Yat-sen University in China and were stored at the School of Life Sciences, Sun Yat-sen University. Sampling information is shown in Table 4.

Currently, dried immature young fruits are widely used as HJH for medicine [88]. The sample processing method and duration were established based on the temperature typically used for drying immature fruits in the local region of Huazhou. All samples underwent drying using a heat pump dryer at a temperature set to 70 °C, humidity 5%, for a period of 7 days.

Additionally, 47 batches of soil samples were air-dried, crushed, sieved, and stored in the laboratory for future use according to the requirements of HJ/T 166.

### 4.2. HS-SPME-GC-MS

The method described by Zheng et al. [17] was modified slightly. Prior to the HS-SPME analysis, the samples were powdered in a mill and passed through a 60-mesh sieve. Then, 20mg of the sample powder was introduced into a 20 mL headspace, adding 5 μL n-tridecane (0.3024 mg/mL dissolved in methanol) serving as an internal standard.

According to the established method [17], GC-MS analysis was carried out using a Trace GC Ultra gas chromatograph coupled with a triple quadrupole mass spectrometer (Agilent Technologies Inc., Palo Alto, CA, USA). Injections were performed in split mode (1:50), and volatile compounds were chromatographed on a HP-5MS column (30 m × 0.25 mm × 0. 25 µm) provided by Agilent Technologies Inc. (Palo Alto, CA, USA), with helium as the carrier gas at a constant flow of 1.0 mL/min. The oven temperature was initially set at 40 °C for 3 min, followed by a gradual increase to 200 °C at a rate of 5 °C/min. This was subsequently increased to 250 °C at a rate of 10 °C/min, and the temperature was then maintained at 250 °C for 3 min. The inlet and ion source temperatures were both set at 230 °C, and MS was scanned at 70 eV in electronic ionization mode. Mass spectra were acquired using the full-scan monitoring mode, covering a range of *m*/*z* 29–448, on an Agilent MassHunter Workstation (NIST17.L, National Institute of Standards and Technology, Gaithersburg, MD, USA). The extraction parameters were set as follows: fiber type 80 μm DVB/C-WR/PDMS-gray, sampling temperature at 50 °C, and a sampling duration of 30 min. Subsequently, the volatile compounds were desorbed in the vaporization chamber at 220 °C for 10 bmin.

For the qualitative analysis, 1 μL of the n-alkane standard mixture was injected under the same analytical conditions as the samples, and the retention times of each n-alkane wre recorded. The retention indices (RIs) of the components were calculated using the Kovats retention index formula. The standard mass spectral database NIST17.L was utilized for retrieval and matching, selecting data with similar retention indices (RI) and a match value exceeding 800.

### 4.3. Climatic Factors

Data for annual mean temperature (BIO1), mean diurnal temperature range (BIO 2), isothermality (BIO 3), temperature seasonality (BIO4), maximum temperature of warmest month (BIO5), minimum temperature of coldest month (BIO6), annual temperature range (BIO7), mean temperature of wettest quarter (BIO8), mean temperature of driest quarter (BIO9), mean temperature of warmest quarter (BIO10), mean temperature of coldest quarter (BIO11), annual precipitation (BIO12), precipitation in the wettest month (BIO13), precipitation in the driest month (BIO14), precipitation seasonality (BIO15), Precipitation in the wettest quarter (BIO16), precipitation in the driest quarter (BIO17), precipitation in the warmest quarter (BIO18), and precipitation in the coldest quarter (BIO19) were obtained from the WorldClim global climate and weather database (http://www.worldclim.org) for the various sampling points. WorldClim is a global database offering high-spatial-resolution weather and climate data, extensively used in the ecological research of medicinal plants [89,90,91].

### 4.4. Soil Elemental Analysis

The soil samples were initially pretreated using a Super Microwave Platform (Ultra WAVE, Milestone, Italy) and were then analyzed for their concentrations of Al, B, Ca, Cu, Fe, K, Mg, Mn, Na, P, S, and Zn via inductively coupled plasma optical emission spectrometry (ICP-OES, ICAP6500 Duo, Thermo Fisher Scientific, Waltham, MA, USA).

In this process, 50 mg of soil was precisely weighed and placed in a polytetrafluoroethylene digestion tube and 4 mL of concentrated nitric acid was added and allowed to pre-digest overnight. On the subsequent day, microwave digestion was conducted. After completion of digestion, the solution was cooled to room temperature, diluted to 10 mL with high-purity water, and designated as the stock solution. Subsequently, the stock solution was diluted 100-fold with 5% diluted nitric acid for analysis.

As in previous studies [38], the operational parameters of the Super Microwave Platform(Ultra WAVE, Milestone, Italy) included pre-pressurizing to 50 bar in advance. Subsequently, the temperature was ramped from room temperature to 90 °C over 10 min, followed by a 5 min equilibrium; then, the temperature was raised from 90 °C to 160 °C over 15 min, with a 5 min equilibrium, then increased from 160 °C to 240 °Cover an additional 15 min, followed by a 30 min equilibrium.

The operational parameters of the Inductively Coupled Plasma Optical Emission Spectrometer(Thermo Fisher Scientific, Waltham, MA, USA) were as follows: RF power set at 1150 W; auxiliary gas flow rate maintained at 0.5 L/min; nebulizer flow rate at 0.5 L/min; and cooling gas flow rate at 12 L/min. Each sample was measured three times to ensure repeatability, and the average value was subsequently calculated.

### 4.5. Data Analysis and Statistics

Qualitative and Quantitative Analysis: Qualitative analysis was performed using the Agilent MassHunter Workstation(Agilent Technologies Inc., Palo Alto, CA, USA) to automatically deconvolute the total ion chromatogram (TIC) of volatile compounds in HJH. The mass spectrometry results were cross-referenced against the 2017 NIST 17.L standard spectral library, applying a filter for substances with a score exceeding 80. The standard mass spectral database NIST17.L was utilized for retrieval and matching, selecting data with similar retention indices (RIs) and match values exceeding 800. For relative quantitative analysis, the ratio of the peak area of each compound to the peak area of the internal standard (n-tridecane) was calculated, which served as an indicator for the abundance of volatile components. This allowed the comparison of compositional variations among HJH samples sourced from different production regions.

The data obtained were primarily processed using R 4.4.1 packages for principal component analysis (PCA), hierarchical cluster analysis (HCA), orthogonal partial least squares discriminant analysis (OPLS-DA), Pearson correlation, multiple linear regression, variance decomposition analysis, random forest, and structural equation modeling (SEM). The Variable Importance in Projection (VIP) and Log_2_FC values were also calculated and predicted. Additionally, statistical significance was assessed using GraphPad Prism 8.0.1, where the selection criteria for volatile compounds included *p* < 0.05, VIP > 1, and Log_2_FC > 1. Differences in soil element data among soil element groups were analyzed and visualized using GraphPad Prism 8.0.1.

## 5. Conclusions

The volatile compounds of HJH are primarily monoterpenes or sesquiterpenes. Differences in the volatile compounds between authentic and non-authentic producing regions were observed, with eight materials of variable quality identified, all of which were significantly higher in samples from authentic regions. This variation might be one of the key factors contributing to confirming the authenticity of HJH. The differential components of volatile compounds across regions included three monoterpenes—β-pinene, α-terpineol, and terpinen-4-ol, the latter two being oxygenated monoterpenoid alcohols—and five sesquiterpenes, namely γ-cadinene, β-copaene, γ-muurolene, bicyclogermacren, and β-cadinene. Modern pharmacological research has shown that β-pinene, γ-cadinene, terpinen-4-ol, and α-terpineol have significant antitussive and expectorant effects [11,12,13,64,65]. The other four compounds, having similar structures, might also possess comparable therapeutic properties. Therefore, HJH from authentic regions might have higher medicinal value, though further validation is required. Additionally, the unique geological and climatic conditions of Huazhou, including climatic factors and soil elements, have been shown to influence the volatile compounds in HJH. In conclusion, the authenticity of HJH is shaped by the distinctive climate and soil composition of Huazhou.

## Figures and Tables

**Table 2:** Identification of prevalent volatile compounds in HJH.

No. ^a^	RT/min	Compound	Relative Area ^b^	
			COREs (*n* = 25)	Non-COREs (*n* = 22)
1	11.97	Pseudolimonene	0.49 ± 0.34	0.09 ± 0.27
2	12.36	β-Pinene	5.27 ± 3.07	0.27 ± 0.18
3	13.65	D-Limonene	25.55 ± 12.94	7.80 ± 8.85
4	14.15	3-Carene	0.14 ± 0.18	0.25 ± 0.52
5	14.55	γ-Terpinene	5.54 ± 5.75	0.30 ± 0.97
6	14.93	Ethyl 2-(5-methyl-5-vinyltetrahydrofuran-2-yl) propan-2-yl carbonate	0.56 ± 0.74	0.20 ± 0.18
7	15.43	Linalool oxide	0.28 ± 0.27	0.10 ± 0.08
8	15.82	Linalyl acetate	0.09 ± 0.05	0.02 ± 0.04
9	18.29	Terpinen-4-ol	0.20 ± 0.08	0.04 ± 0.06
10	18.72	α-Terpineol	0.19 ± 0.08	0.04 ± 0.05
11	24.95	2-methylene-4,8,8-trimethyl-4-vinyl-bicyclo[5.2.0]nonane	2.22 ± 0.82	0.71 ± 1.14
12	25.86	1,5,9,9-Tetramethyl-1,4,7-cycloundecatriene	0.27 ± 0.07	0.10 ± 0.13
13	26.32	γ-Cadinene	1.09 ± 0.29	0.23 ± 0.25
14	26.52	β-Copaene	15.16 ± 4.28	3.61 ± 4.51
15	26.76	γ-Muurolene	0.68 ± 0.16	0.18 ± 0.19
16	26.86	Bicyclogermacren	1.73 ± 0.45	0.46 ± 0.53
17	27.37	β-Cadinene	1.43 ± 0.36	0.37 ± 0.39

^a^ Numbers represent the compound peaks for volatile compounds of HJH. ^b^ Peak–area ratios relative to n-Tridecane (internal standard).

**Table 3:** Environmental factors.

Types	No.	Description
Temperature	BIO1	Annual Mean Temperature
	BIO5	Max Temperature of Warmest Month
	BIO6	Min Temperature of Coldest Month *
	BIO8	Mean Temperature of Wettest Quarter
	BIO9	Mean Temperature of Driest Quarter
	BIO10	Mean Temperature of Warmest Quarter
	BIO11	Mean Temperature of Coldest Quarter
Temperature variation	BIO2	Mean Diurnal Range
	BIO3	Isothermality
	BIO4	Temperature Seasonality
	BIO7	Temperature Annual Range *
Precipitation	BIO12	Annual Precipitation
	BIO13	Precipitation of Wettest Month
	BIO14	Precipitation of Driest Month
	BIO16	Precipitation of Wettest Quarter
	BIO17	Precipitation of Driest Quarter
	BIO18	Precipitation of Warmest Quarter *
	BIO19	Precipitation of Coldest Quarter
Precipitation variation	BIO15	Precipitation Seasonality *
Soil	1	Al *
	2	B *
	3	Ca *
	4	Cu *
	5	Fe *
	6	K *
	7	Mg *
	8	Mn *
	9	Na *
	10	P
	11	S
	12	Zn *

* Represents the environmental factors brought into the model after screening.

**Table 4:** Sampling information.

Province	Samples	Longitude (°)	Latitude (°)	Altitude (m)
Guangdong	Non-CORE 1	110.417773	21.821382	64.0
Guangdong	Non-CORE 2	114.005000	25.004000	156.0
Hunan	Non-CORE 3	109.706450	28.135200	308.0
Guangxi	Non-CORE 4	110.613407	22.847995	119.0
Jiangxi	Non-CORE 5	115.311141	27.167075	80.0
Guangdong	Non-CORE 6	116.331538	24.550424	187.9
Guangdong	Non-CORE 7	116.328331	24.549561	194.0
Guangdong	Non-CORE 8	116.329475	24.551128	183.9
Jiangxi	Non-CORE 9	116.314680	29.556333	52.0
Hunan	Non-CORE 10	110.986500	27.170280	292.0
Jiangxi	Non-CORE 11	113.966709	26.748899	230.3
Jiangxi	Non-CORE 12	113.966291	26.748526	229.4
Zhejiang	Non-CORE 13	120.757162	30.126701	24.5
Hunan	Non-CORE 14	111.625134	29.717790	52.6
Jiangxi	Non-CORE 15	116.296923	29.513210	34.0
Sichuan	Non-CORE 16	104.726995	30.369177	403.0
Sichuan	Non-CORE 17	104.726995	30.369177	403.0
Sichuan	Non-CORE 18	104.726995	30.369177	403.0
Guizhou	Non-CORE 19	108.220000	28.110000	581.0
Jiangxi	Non-CORE 20	114.686367	25.650904	157.7
Jiangxi	Non-CORE 21	114.690331	25.647292	160.0
Jiangxi	Non-CORE 22	114.693850	25.681852	145.2
Huazhou	CORE 1	110.404632	21.997887	40.1
Huazhou	CORE 2	110.355038	22.016040	42.2
Huazhou	CORE 3	110.412663	22.043070	39.3
Huazhou	CORE 4	110.419240	22.015369	41.1
Huazhou	CORE 5	110.402059	21.980484	55.6
Huazhou	CORE 6	110.354744	22.016749	45.0
Huazhou	CORE 7	110.418387	22.003365	94.1
Huazhou	CORE 8	110.413502	22.005701	49.1
Huazhou	CORE 9	110.408349	22.103947	81.7
Huazhou	CORE 10	110.425757	22.177994	77.8
Huazhou	CORE 11	110.388222	22.092638	52.3
Huazhou	CORE 12	110.568212	21.983016	27.6
Huazhou	CORE 13	110.552141	22.114187	77.2
Huazhou	CORE 14	110.660837	22.140085	43.6
Huazhou	CORE 15	110.417253	21.820815	55.4
Huazhou	CORE 16	110.414948	21.989426	56.0
Huazhou	CORE 17	110.478683	21.693335	22.1
Huazhou	CORE 18	110.481566	21.693754	31.6
Huazhou	CORE 19	110.458909	21.696219	54.1
Huazhou	CORE 20	110.659785	21.752218	15.4
Huazhou	CORE 21	110.659428	21.751407	22.7
Huazhou	CORE 22	110.577771	21.786409	32.5
Huazhou	CORE 23	110.518970	21.939292	19.8
Huazhou	CORE 24	110.573151	21.665990	6.8
Huazhou	CORE 25	110.622842	21.854864	39.1

## Data Availability

Data are contained within the article.
